# Overexpression of a Mitogen-Activated Protein Kinase *SlMAPK3* Positively Regulates Tomato Tolerance to Cadmium and Drought Stress

**DOI:** 10.3390/molecules24030556

**Published:** 2019-02-03

**Authors:** Tayeb Muhammad, Jie Zhang, Yalin Ma, Yushun Li, Fei Zhang, Yan Zhang, Yan Liang

**Affiliations:** 1 College of Horticulture, Northwest A&F University, Yangling 712100, China; tayebmuhammad@nwsuaf.edu.cn (T.M.); 15591858981@163.com (J.Z.); 15891785931@163.com (Y.M.); liyushunaf@163.com (Y.L.); feizhang@nwsuaf.edu.cn (F.Z.); 2 State Key Laboratory of Crop Stress Biology in Arid Regions, Northwest A&F University, Yangling 712100, China

**Keywords:** antioxidant enzymes, cadmium, drought, lipid peroxidation, reactive oxygen species, *SlMAPK3*, tomato

## Abstract

Mitogen-activated protein kinases (MAPKs) activation is a common defense response of plants to a range of abiotic stressors. *SlMPK3*, a serine-threonine protein kinase, has been reported as an important member of protein kinase cascade that also functions on plant stress tolerance. In this study, we cloned *SlMPK3* from tomato and studied its role in cadmium (Cd^2+^) and drought tolerance. The results showed that transcripts of *SlMAPK3* differentially accumulated in various plant tissues and were remarkably induced by different abiotic stressors and exogenous hormone treatments. Overexpression of *SlMAPK3* increased tolerance to Cd^2+^ and drought as reflected by an increased germination rate and improved seedling growth. Furthermore, transgenic plants overexpressing *SlMAPK3* showed an increased leaf chlorophyll content, root biomass accumulation and root activity under Cd^2+^ stress. Chlorophyll fluorescence analysis revealed that transgenic plants demonstrated an increased photosynthetic activity as well as contents of chlorophyll, proline, and sugar under drought stress. Notably, cadmium- and drought-induced oxidative stress was substantially attenuated in *SlMAPK3* overexpressing plants as evidenced by lower malondialdehyde and hydrogen peroxide accumulation, and increased activity and transcript abundance of enzymatic antioxidants under stress conditions compared to that of wild-type. Our findings provide solid evidence that overexpression of *SlMAPK3* gene in tomato positively regulates tolerance to Cd^2+^ and drought stress, which may have strengthen the molecular understanding of *SlMAPK3* gene to improve abiotic stress tolerance.

## 1. Introduction

Plants are sessile living organisms, often exposed to diverse biotic and abiotic stress conditions throughout their life cycles. Abiotic stress not only affects plant growth, yield, and quality but also limits the geographical distribution of many plants [1]. While most abiotic stressors occur naturally, human activities may change the abundance and severity of some stressors, such as heavy metals. Heavy metal toxicity is one of the serious environmental problems that affect both plants, animals and public health [2].

In plants, Cd^2+^ affects many physiological and metabolic processes by inducing oxidative stress [3]. High concentrations of Cd^2+^ disrupt membrane integrity and cause loss of function of the plasma membrane [4], chromosomal aberration, and leaf chlorosis [5,6]. Furthermore, toxic concentrations of Cd^2+^ can reduce seed germination, inhibit plant growth and cause wilting or even plant death [7,8,9]. Similarly, drought stress has profound detrimental effects on seed germination, plant growth, and crop productivity. Drought causes stomatal closure and reduces the activity and contents of the photosynthetic enzymes in carbon reduction cycle, including, ribulose-one, five-bisphosphate carboxylase/oxygenase [10]. On the other hand, plants have developed a variety of mechanisms to sense and cope with the unfavorable environmental conditions. Multiple signal transduction pathways function in concert to mediate plant adaptation to hostile environments [11]. One of the most important signaling modules that deliver perceived external stimuli to the nucleus is the mitogen-activated protein kinase (MAPK) cascade [12].

MAPK cascades regulate many essential biological processes, including growth, development and programmed cell death (PCD) in plants [13]. These are three-tiered phospho-relay cascade consisting of MAPKK kinases that activate via phosphorylation of their downstream MAPKKs, which in turn further activate MAPKs [14]. MAPKs in plants are classified into four different homologous groups (A, B, C, and D) on the basis of their sequence and conserved motifs [15], with members of families A and B being particularly involved in hormonal and environmental responses [16]. A total of 20 *AtMAPKs* and 16 *SlMAPKs* occur in *Arabidopsis* and tomato, respectively, and the transcript levels of nearly all *SlMAPKs* genes are significantly increased by stress [17].

The upstream kinases activate downstream kinases to activate various physiological functions, and it has been reported that reactive oxygen species (ROS), phosphatidic acid, and various hormones are also involved in the activation of MAPK cascade [18,19,20]. Previous reports have shown that MKK9 activates MPK3/MPK6 to regulate the ethylene biosynthesis and signaling, leaf senescence and phosphate acquisition [21,22,23,24]. In *Arabidopsis,* the *AtMKK2* phosphorylates *AtMPK4* and *AtMPK6* (downstream kinases) after being stimulated by cold and salt stress [25], whereas mechanical wounding [26] and osmotic stress activate MAPK3 [27]. The MAPK cascades also receive various signals to induce resistance against different biotic stressors. In *Nicotiana tabacum* (tobacco) two MAPKs, salicylic acid (SA) induced protein kinase (*NtSIPK*) homologous to *AtMPK6* and wounding induced protein kinase (*NtWIPK*) homologous to *AtMPK3* in *Arabidopsis*, are activated in an N-gene-dependent manner to mount local and systemic antiviral defense responses [28]. Ethylene, salicylic acid, and MAPK-related defense signaling pathways enhance resistance to bacterial wilt, and the induction of plant aminocyclopropane carboxylic acid oxidase (ACO) gene family occurs through MPK1-, 2, and 3 pathways following diethyl ether (ether) treatment [29,30].

Tomato *LeMPK1, LeMPK2*, and *LeMPK3* are activated in response to fungal toxin fusicoccin [31], and have roles in hypersensitive response (HR) and resistance [32]. Similarly *LeMPK1, LeMPK2,* and *LeMPK3* are induced by ultraviolet-B (UV-B) radiations and the dual specificity of *LeMPK3* characterizes a convergence point for numerous signaling pathways that induce defense responses [33]. In addition, previous results reveal that *SpMPK3* influences stomatal activity and hydrogen peroxide (H_2_O_2_) accumulation through the abscisic acid (ABA)-H_2_O_2_ pathway to enhance drought tolerance in tomato and also functions in *Arabidopsis* seed germination and seedling development during osmotic stress [34,35].

Cultivated tomato is susceptible to a wide range of stressors and researchers have long been studying potential genes for resistance in plants. However, the gene expression, signal transduction, and cellular responses to stress remain unclear, under Cd^2+^ stress. Thus, considering the importance of MAPK3 in stress tolerance, we cloned *SlMAPK3* gene to investigate its functions against cadmium and drought stresses. Our results showed that overexpression of *SlMAPK3* enhanced tolerance to Cd^2+^ and drought stress by improving multiple morphological and physiological characters.

## 2. Results

### 2.1. Characterization of SlMAPK3 Gene

We isolated *SlMPK3* from tomato using specific primers. The *SlMPK3* cDNA contains an 1122-bp open reading frame (ORF) with a total length of 1657-bp long. The untranslated regions are 129-bp and 406-bp at 5′ and 3′ ends, respectively (https://www.ncbi.nlm.nih.gov/nuccore/NM001247431). Tomato genome database showed that the *SlMAPK3* gene is located on chromosome 6, encoding a protein of 373 amino acids having approximately 42.66 kDa molecular weight and an iso electric point 5.25. Tomato MAPKs family contains 16 genes [17]. On the basis of phylogenetic analysis, they are divided into four groups and MAPK3 belongs to the group A having two other members of the family (Figure S1A). Amino acid analysis showed that MAPKs family contains Serine/Threonine protein kinases catalytic domain. Furthermore, the catalytic domain of *SlMAPK3* consists of total 288 amino acids (Figure S1B).

### 2.2. Expression Analysis of SlMAPK3

**Figure 1:**
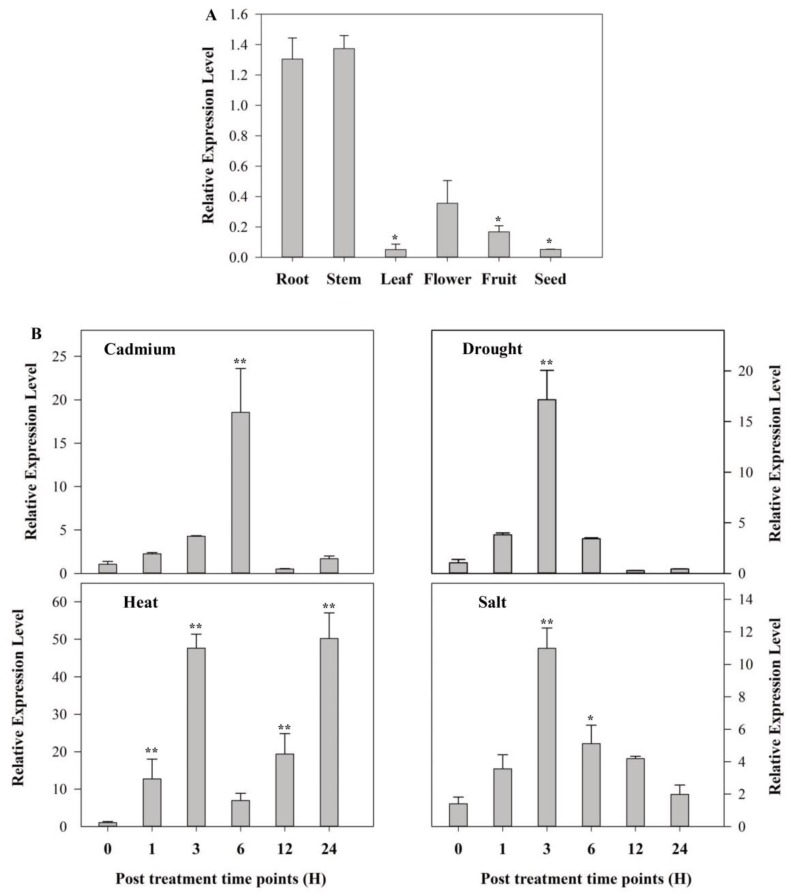
Transcript levels of *SlMAPK3* in tomato as influenced by different tissues types and abiotic stressors. (**A**) *SlMAPK3* expression in different plant tissues, and (**B**) time course of *SlMAPK3* transcript under abiotic stresses induced by cadmium (150 μM CdCl_2_ 2.5H_2_O), drought (dehydration), heat (40 °C) and salt (200 mM NaCl). Data represent mean ± SE of three biological replicates. **, * significant level differed at *p* < 0.01 and *p* < 0.05, respectively.

Tissue-specific expression analysis revealed that transcript levels of the *SlMAPK3* were higher in stems, roots, and flowers as compared to other tissues of *Solanum lycopersicum* (Figure 1A). To understand the role of *SlMAPK3*, the expression pattern of *SlMAPK3* was studied in response to various abiotic stressors and hormonal treatments. Real-time reverse transcription–PCR (RT–PCR) analysis revealed that the transcript levels of *SlMAPK3* increased in response to cadmium, dehydration, salt, heat, ABA, methyl jasmonate (MeJA) and SA treatments. *SlMAPK3* was gradually induced by cadmium, drought and salt treatments, reaching the maximum levels following 6, 3 and 3 h exposure, respectively. However, in case of the heat stress, the expression levels of *SlMAPK3* reached the highest peak after 24 h exposure (Figure 1B). Hormonal application rapidly up-regulated expression levels of *SlMAPK3,* which peaked after 1 h treatment and then declined (Figure S2).

### 2.3. Identification of Transgenic Plants

**Figure 2:**
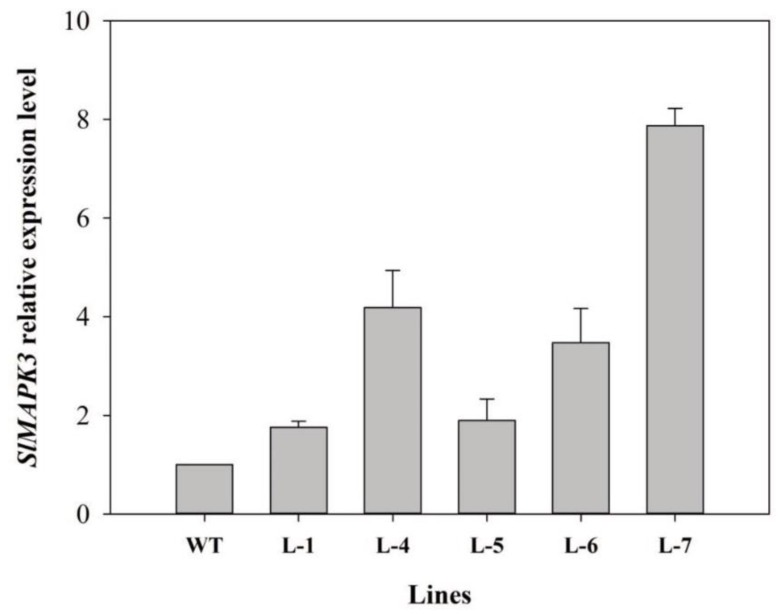
Expression levels of SlMAPK3 in wild-type and transgenic lines. Expression data were normalized with SlMAPK3 expression in WT as 1. Error bars show the standard error between three replicates.

To generate transgenic plants, the full coding sequence of *SlMAPK3* was cloned in to binary vector under the control of cauliflower mosaic virus (CaMV) 35S promoter and the resultant vector was transfer through *Agrobacterium tumefaciens* mediated transformation. The transgenic lines were screened for kanamycin resistance and then confirmed by genomic PCR (Figure S3). The relative expression of five homozygous lines were normalized to the wild-type expression level (wild-type = 1.0) and qRT-PCR analysis revealed that transgenic lines exhibited high expression levels than wild-type (Figure 2). The three independent lines, L-4, L-6, L-7, with relatively high expression levels were selected for further experiments.

### 2.4. Growth Analysis of Seedlings Under Cadmium Stress

**Figure 3:**
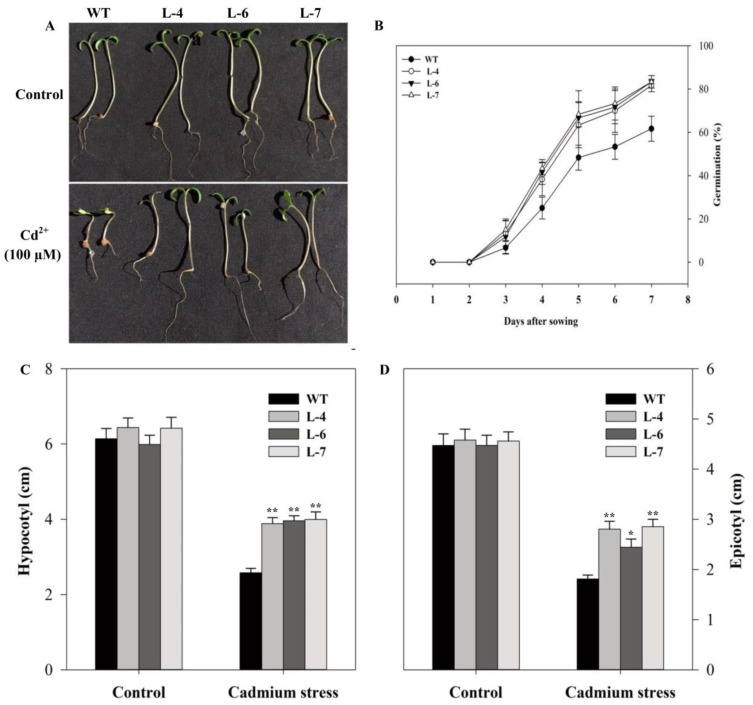
Seed germination and seedling growth of wild-type and *SlMAPK3* overexpressed plants under cadmium stress. (**A**) Seedling phenotypes in the medium supplemented with 0 and 100 µM cadmium. (**B**) Seed germination percentage under cadmium stress. (**C**) Hypocotyl length and (**D**) epicotyl length in control and cadmium stress conditions. Data represent mean ± SE of three biological replicates. For C and D, **, * significant level differed at *p* < 0.01 and *p* < 0.05, respectively.

To study the possible role of *SlMAPK3* gene in cadmium tolerance, both wild-type (WT) and transgenic lines were germinated on ½ strength Murashige and Skoog (MS) medium supplemented with 0 and 100 µM Cd^2+^. No substantial difference was observed between transgenic and WT lines at control condition. However, *SlMAPK3*-overexpressing lines showed significantly higher germination rate than WT when grown on the Cd^2+^ contaminated medium than WT (Figure 3B). We also investigated the growth of seedling under the same conditions by measuring the newly developed stem and the root of the seedlings. While no significant change was found between WT and three transgenic lines under the unstressed condition, the growth of hypocotyl and epicotyl increased by 57.6% and 49.81%, respectively in transgenic plants as compared to that in WT plants under Cd^2+^ stress (Figure 3C,D). These findings indicate that *SlMAPK3* overexpression enhanced tomato tolerance to cadmium stress during seed germination.

### 2.5. SlMPK3 Overexpression Improves Plant Tolerance to Cd^2+^ Stress

**Figure 4:**
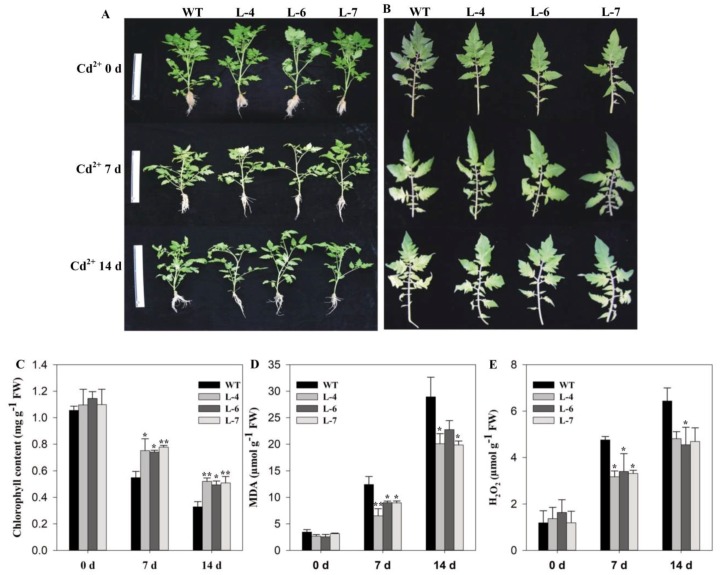
Overexpression of *SlMAPK3* enhanced cadmium tolerance in tomato. (**A**) Plant phenotypes. (**B**) Images of the second leaves of WT and *SlMAPK3* overexpressed lines (**C**) chlorophyll content, (**D**) malondialdehyde (MDA) content, and (**E**) H_2_O_2_ accumulation. Seedlings of WT and transgenic lines at five leaf-stage were treated with 0 (0 days) and 100 µM (7- and 14-days) cadmium. For **C**–**E**, data represent mean ± SE of three biological replicates. **, * significant level differed at *p* < 0.01 and *p* < 0.05, respectively.

To further evaluate the possible role of *SlMAPK3* in plant tolerance to cadmium stress, tomato plants at five-leaf-stage were subjected to Cd^2+^ stress. After 7- and 14-days stress treatments, chlorosis was observed in leaves of both transgenic and WT seedlings, but the symptoms were more severe in WT as compared to that in overexpressed lines. On the other hand, no chlorosis in leaves was found in all lines under the control condition (Figure 4B). In line with the visual observation, spectrophotometric analysis also showed that Cd^2+^ stress significantly reduced chlorophyll content in leaves. Compared with the transgenic lines, the chlorophyll content decreased by 38.01% and 17.9% in control plants following 7- and 14-days Cd^2+^ treatments, respectively (Figure 4C). Under control condition, malondialdehyde (MDA) and H_2_O_2_ contents remained similar in all plants. However, Cd^2+^ treatment resulted in a remarkable increase in MDA and H_2_O_2_, but the levels were significantly low in the overexpressed lines compared to that in the WT (Figure 4D,E). The MDA content of the WT plants increased 3.59 and 8.38 fold at 7- and 14-days after Cd^2+^ treatment, respectively, while in transgenic lines MDA content was significantly low compared to WT (Figure 4D). H_2_O_2_ accumulation also increased with the progression of Cd^2+^ stress, however, the accumulation of H_2_O_2_ was maximum in WT as compared to that in transgenic lines (Figure 4E).

**Figure 5:**
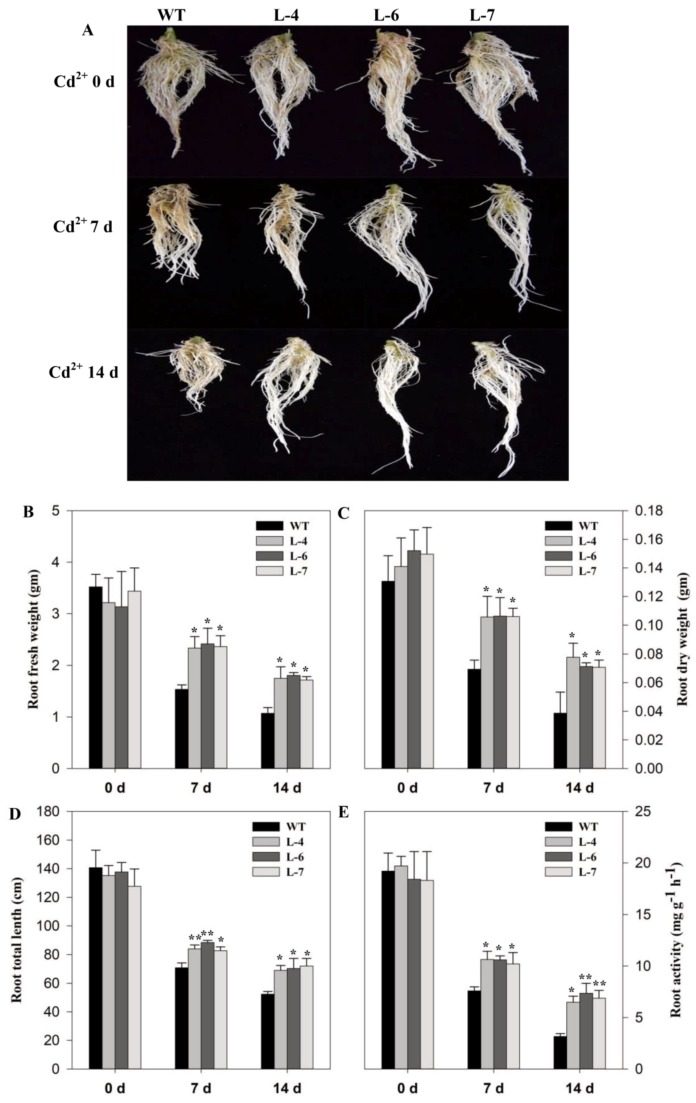
Overexpression of *SlMAPK3* improved root morphology under cadmium stress in tomato. (**A**) Roots phenotype, (**B**) root fresh weight, (**C**) root dry weight, (**D**) root total length, and (**E**) root activity. Seedlings of WT and transgenic lines at five leaf-stage were treated with 0 (0 days) and 100 µM (7- and 14-days) cadmium. For **B**–**E**, data represent mean ± SE of three biological replicates. **, * significant level differed at *p* < 0.01 and *p* < 0.05, respectively.

Cd^2+^ toxicity relates well with the inhibition of root growth, therefore, we studied the root morphology of WT and transgenic lines in control and Cd^2+^ stress conditions. It was observed that there was no change in root morphology in control condition, however, Cd^2+^ stress for 7- and 14-days inhibited root growth in tomato genotypes but the maximum inhibition was found in WT as compared to transgenic lines (Figure 5A). From the measurements of the roots for different characteristics, we found that Cd^2+^ stress severely affected WT plants, as evidenced by the root weight and total length. Root fresh weight and dry weight were significantly higher in overexpressed plants than WT subjected to stress conditions (Figure 5B,C). Total root length was also significantly reduced in WT plants as compared to transgenic lines following Cd^2+^ stress (Figure 5D). Analysis of root activity showed a significant reduction after Cd^2+^ stress. At 14-days post Cd^2+^ stress, the root activity of the WT plants was 2.18 fold lower than the average root activity of transgenic lines (Figure 5E).

### 2.6. SlMPK3 Overexpression Enhances Enzymatic Antioxidant Potential under Cd^2+^ Stress

**Figure 6:**
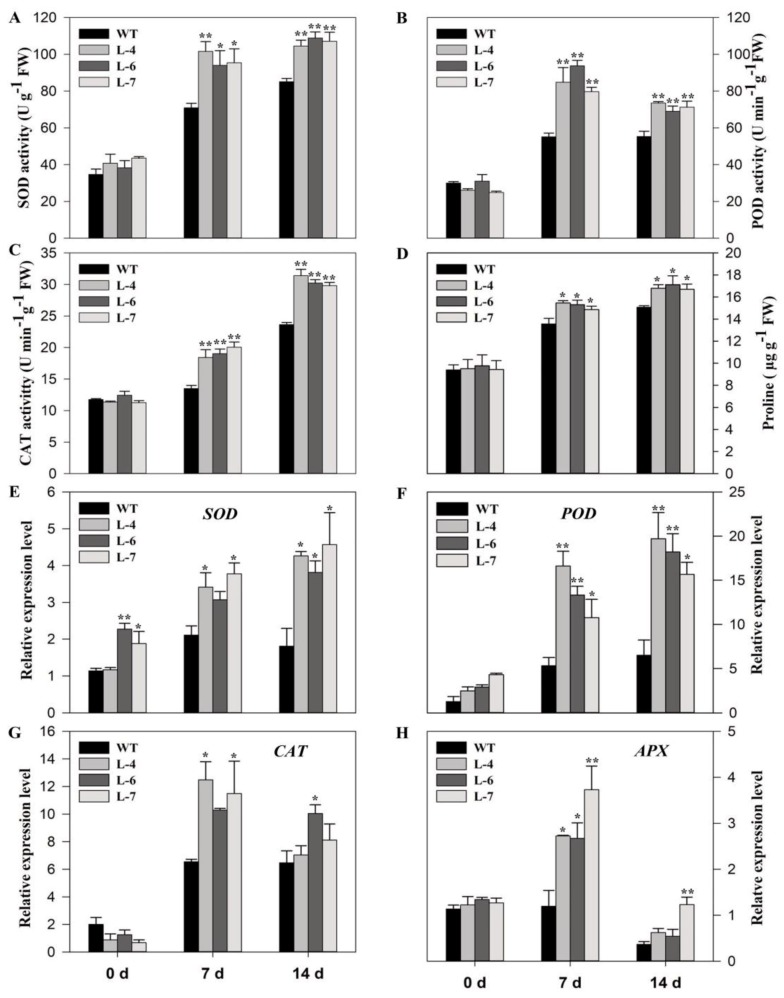
Antioxidant enzymes activities and transcript level of related genes in WT and transgenic lines under cadmium stress. (**A**–**C**) Activities of SOD, POD and CAT respectively. (**D**) Proline content and expression analysis of (**E**) *SlSOD*, (**F**) *SlPOD*, (**G**) *SlCAT*, and (**H**) *SlAPX*. Seedlings of transgenic lines and WT at five leaf stage were treated with 0 (0 days) and 100 µM (7- and 14-days) cadmium. Data represent mean ±SE of three biological replicates. **, * significant level differed at *p* < 0.01 and *p* < 0.05, respectively.

Since stress conditions promote ROS accumulation, plants activate the antioxidant system to scavenge overproduced ROS and minimize ROS-induced damage. Therefore, antioxidant enzymes activities and the transcript levels of relevant genes were measured in both WT and transgenic lines after exposure of plants to Cd^2+^ stress for 7- and 14-days. Under normal condition, there were no observable differences between wild-type and *SlMPK3*-overexpressed lines regarding antioxidant enzymes activity and proline content (Figure 6A–D). 

However, Cd^2+^ treatment significantly increased the superoxide dismutase (SOD) and catalase (CAT) activities at both at 7- and 14-day post treatments (Figure 6A,C), while, peroxidase (POD) activity initially increased at 7-days and then decreased at 14-days post treatment in all tomato genotypes (Figure 6B). Cd^2+^ treatment also increased the levels of proline in both transgenic and WT plants, but the elevation was significantly higher in the overexpressed lines after 7- and 14-days of Cd^2+^ stress (Figure 6D). Overall, the antioxidant activity was significantly higher in transgenic lines than that in WT.

To further explore this mechanism, the transcript levels of antioxidant-related genes were studied in control and Cd^2+^-stressed plants. The expression of *SlSOD* and *SlPOD* increased at 7- and 14-days after Cd^2+^ stress compared to the control that were not treated with Cd^2+^; however, the expression of *SlSOD* and *SlPOD* remained higher in the three overexpressed tomato lines than in the WT (Figure 6E,F). The expression of *SlCAT* and *SlAPX*, in the over-expressing lines initially increased at 7-days and then declined at 14-days (Figure 6G,H).

### 2.7. Assessment of Drought Stress Tolerance in SlMAPK3-Overexpressed and Wild-Type Plants

**Figure 7:**
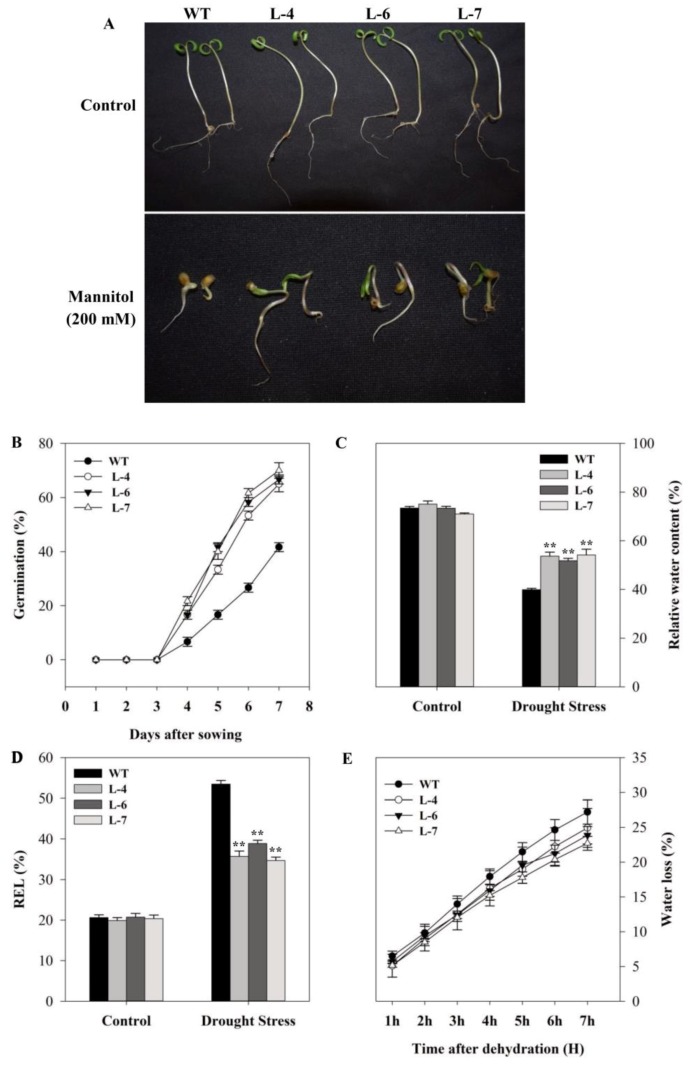
*SlMAPK3* overexpression enhanced drought tolerance in tomato. (**A**) Seedling growth in 0 and 200 mM mannitol stress, (**B**) seed germination percentage under 200 mM mannitol stress, (**C**) relative water content, (**D**) relative electrolyte leakage, and (**E**) water loss in detached leaves. For **B**–**E**, data represent mean ± SE of three biological replicates. For C and D, ** significant level differed at *p* < 0.01.

To evaluate the drought tolerance capability as influenced by *SlMAPK3* overexpression, first, we tested the seed germination rate in WT and transgenic lines following treatment with mannitol (200 mM) supplemented medium. There were no observable differences in seedling length and germination rate between the transgenic and WT seedlings in a medium without mannitol (Figure 7A,B). However, in the presence of mannitol, the germination rate of WT plants decreased by approximately 61.35% compared with the transgenic lines (Figure 7B). There were no significant differences in leaf relative water content (RWC) and relative electrolyte leakage (REL) between overexpressed and wild-type plants under well-watered condition. After exposure of plants to drought stress for 15 days, water content and electrolyte leakage increased and decreased by 33.23% and 46.70%, respectively in transgenic lines compared with that of WT plants, indicating that drought stress-caused membrane damage was severe in WT plants (Figure 7C,D). For further confirmation, water loss assay was performed with detached leaves. It was observed that the rate of water loss was faster and higher (by 14.14%) in WT than that of *SlMAPK3*-overexpressing transgenic plants (Figure 7E).

### 2.8. Overexpression of SlMAPK3 Minimizes H_2_O_2_ Accumulation and Improves Photosynthetic Activity

**Figure 8:**
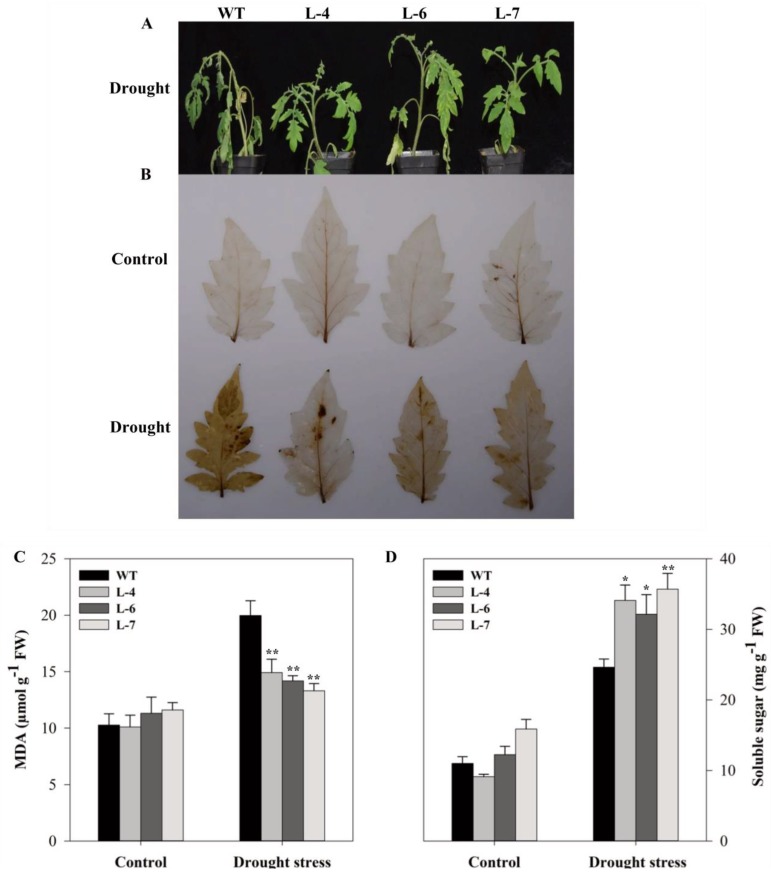
Overexpression of *SlMAPK3* alleviated drought-induced oxidative stress in tomato. (**A**) Seedling phenotypes, (**B**) DAB staining for visualization of H_2_O_2_ accumulation, (**C**) MDA content, and (**D**) soluble sugar content. Six-week old seedlings of WT and transgenic lines were subjected to drought stress by withholding water supply for 15 successive days and well-watered plants (watered every alternate day) served as a control. For **C** and **D** data represent mean ± SE of three biological replicates. **, * significant level differed at *p* < 0.01 and *p* < 0.05, respectively.

The transgenic and WT lines were exposed to dehydration stress for 15 days, and it was found that wilting of leaves was faster and more severe in WT plants than that of overexpressed plants (Figure 8A). Diaminobenzidine (DAB) staining showed no obvious difference between different lines under normal conditions. However, drought stress increased H_2_O_2_ accumulation, more profoundly in WT, as evidenced by the relatively intense dark brown color deposits compared to the normal condition (Figure 8B). Next, MDA content was measured as an indicator of lipid peroxidation. There were no apparent differences in the levels of this compound under normal conditions. However, drought stress significantly altered the levels of malondialdehyde in overexpressed and wild-type plants. The MDA content in three transgenic lines was approximately 41.40% lower than that of WT after drought stress (Figure 8C). Soluble sugar content showed no statistical variations between control and transgenic plants (except L-7) under normal condition. After drought treatment, soluble sugar content increased in all lines, however, *SlMAPK3* overexpressing plants accumulated 37.93 % higher soluble sugar than WT (Figure 8D).

**Figure 9:**
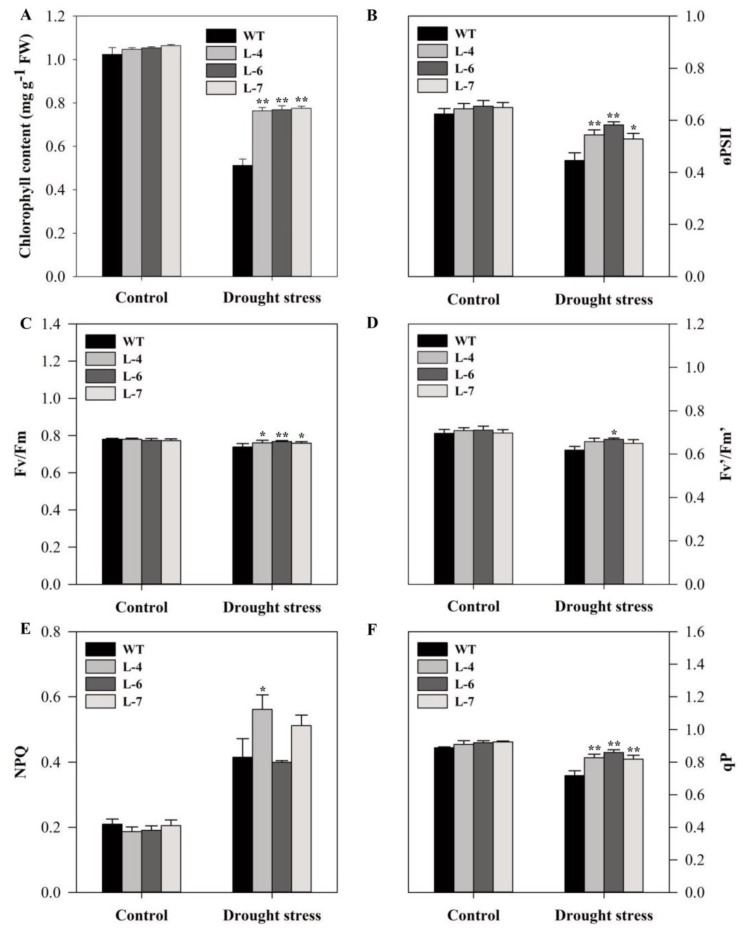
Overexpression of *SlMAPK3* improved photosynthetic activity under drought stress. (**A**) chlorophyll content, (**B**) quantum efficiency of PSII photochemistry (øPSII), (**C**) the maximum photochemical efficiency of PSII (*Fv/Fm*), (**D**) the efficiency of energy capture by open PSII reaction centers (*Fv’/Fm’*), (**E**) non-photochemical quenching (NPQ), and (**F**) photochemical quenching (qP). Two-months-old seedlings of WT and transgenic lines were subjected to drought stress by withholding water supply for 20 successive days and well-watered plants (watered every alternate day) served as control. Data represent mean ±SE of three biological replicates. **, * significant level differed at *p* < 0.01 and *p* < 0.05, respectively.

Meanwhile, drought stress decreased chlorophyll content by 25.70% in WT plants as compared to overexpressing plants, while, no difference was found between them under irrigated condition (Figure 9A). We also analyzed some chlorophyll fluorescence parameters in two-month-old plants that were subjected to 20-days drought condition. The differences were not significant between WT and transgenic plants in terms of chlorophyll fluorescence parameters at well-watered conditions except for qP in L-6 and L-7 (Figure 9B–F). However, drought stress decreased quantum efficiency of PSII photochemistry (øPSII), the maximum photochemical efficiency of PSII (*Fv*/*Fm*), the efficiency of energy capture by open PSII reaction centers (*Fv’*/*Fm’*) and the efficiency of photochemical quenching (qP) in both WT and overexpressing lines, but WT plants showed a greater decrease compared to that of overexpressing lines (Figure 9B–D and F). In case of non-photochemical quenching (NPQ), the efficiency increased after stress compared to the normal condition, being higher in the L-4 and L-7 than in the WT (Figure 9E).

### 2.9. Analysis of Antioxidant Enzyme Activity and Expression of Related Genes after Drought Stress

**Figure 10:**
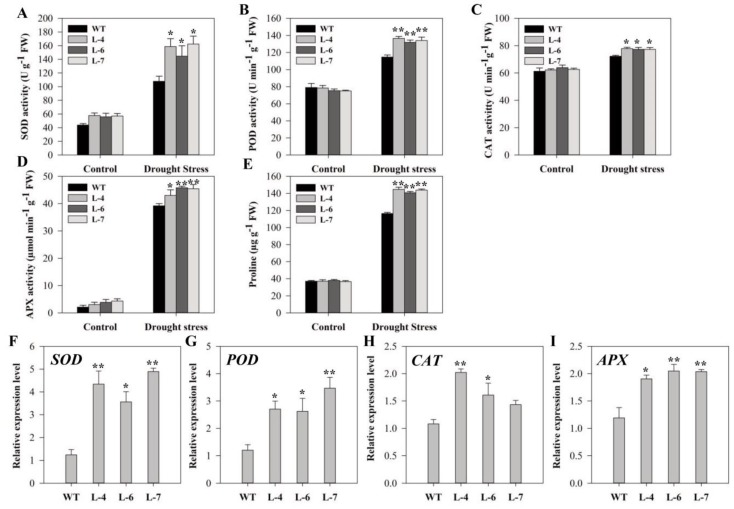
Comparison of enzymatic activity and relative expression of stress-responsive genes in wild-type and transgenic plants under normal and drought stress conditions. (**A**–**D**) Activity of SOD, POD, CAT, and APX, respectively. (**E**) Proline content and expression analysis of (**F**) *SlSOD*, (**G**) *SlPOD*, (**H**) *SlCAT*, and (**I**) *SlAPX*. Six-week old seedlings of WT and transgenic lines were subjected to drought stress by withholding water supply for 15 successive days and well-watered plants (watered every alternate day) served as a control. Data represent mean ± SE of three biological replicates. **, * significant level differed at *p* < 0.01 and *p* < 0.05, respectively.

Plants need an effective antioxidant system to scavenge or neutralize the excess ROS levels, thereby minimizing the oxidative damage during stress conditions. Therefore, the activities of antioxidant enzymes were measured in transgenic and WT seedlings under control and drought conditions. The results showed that the activities of all the antioxidant enzymes and content of proline were statistically similar under normal conditions in all lines except SOD, which was statistically higher in L-4 than that in WT (Figure 10A–E). After drought treatment, activities of those four antioxidant enzymes increased in both WT and transgenic plants, but transgenic lines had significantly higher activities than wild-type. In *SlMAPK3* overexpressing plants, SOD, POD, CAT, and Ascorbate peroxidase (APX) activities, as well as proline content, were 93.96%, 16.91%, 7.2%, 14.22%, and 22.9%, respectively higher than WT (Figure 10A–E). To better understand the molecular mechanisms of *SlMAPK3*–mediated drought tolerance, transcript levels of ROS detoxification -related genes such as *SlSOD, SlPOD, SlCAT,* and *SlAPX* were examined after 15 days of drought stress (Figure 10F–I). qRT-PCR results showed that drought stress significantly induced the expression of all antioxidants related genes in transgenic plants as compared to WT, with the only exception for CAT in L-7, which was not statistically significant (Figure 10F–I).

## 3. Discussion

Since germination to subsequent development, plants face various biotic and abiotic stresses throughout their life cycles. Depending on the severity of the stressors, a stress condition not only affects plant growth and development but also limits agricultural productivity. In the past, traditional breeding methods have been practiced to reduce various stress-related losses but due to the complexity in plant stress tolerance mechanism, only a minimum goal was achieved [36]. Recently, molecular and genetic engineering are being considered as the most effective approaches for the development of stress-tolerant crops. These advanced techniques unfold the physiological and biochemical pathways responsible for natural plant tolerance against stresses. MAPKs cascades are vital for plant growth, development and stress responses [13]. For instance, *OsMPK5* in rice is an ortholog of *AtMPK3* and *NtWIPK* in *Arabidopsis* and tobacco, respectively, can be activated by various biotic and abiotic stressors [37,38]. In addition, to stress-responsive signaling, *AtMPK3* and *AtMPK6*, participate in stomatal patterning and development [39]. Despite a significance number of studies of different biochemical pathways relating to plant stress tolerance, molecular mechanisms that underlie stress perception and responses, especially Cd^2+^ remain poorly understood. Here, we demonstrated that overexpression of *SlMAPK3* enhanced tolerance to Cd^2+^ and drought stress in tomato.

In this study, *SlMAPK3* was cloned and overexpressed in tomato to study its role in plant tolerance to cadmium and drought stress. Tomato *SlMAPK3* is located on chromosome 6, and contains 1122-bp ORF, 373 aa sequence and 5.25 Pls theoretical isoelectric point. This gene is a member of group A and contains a serine/threonine protein kinases catalytic domain. The quantitative real-time PCR (qRT-PCR) analysis showed differential *SlMAPK3* expression in various tissues (Figure 1A), whereas cadmium and drought stress treatment significantly increased transcript levels of *SlMAPK3* (Figure 1B). Previous reports have shown that *SlMPK3* and *SlMPK7* are stimulated by various stress conditions such as drought, low temperature, and signaling molecules [40,41]. Similarly, high concentrations of cadmium ions activate four different MAPKs (SAMAK, SIMK, MMK2 and MMK3) and induce different cellular mechanisms [42]. In rice, Cd^2+^-induce activation of 42 kDa MAP kinase, which shows activity of *OsMPK3* and *OsMPK6* [2], indicating the possible role of *SlMAPK3* in tomato growth, development and stress tolerance.

Notably, exposure of plants to Cd^2+^ stress can cause morphological and physiological changes in the stressed plants. At the morphological level, symptoms of Cd^2+^ toxicity include chlorosis of leaves and inhibition of root growth [43,44]. Cd^2+^ administration induces expression of *OsMAPK2* and significantly decreases root fresh weight and dry weight in rice seedlings [45]. Similarly, in *Arabidopsis*, *AtOXI1* a serine-threonine kinase is induced by various stimuli, which is also required for the activation of *AtMPK-3, -6* and root hair growth [46]. From the previous reports, it can be concluded that serine/threonine kinase has important functions in maintaining root morphology during stress conditions. In the current study, we found that seed germination rate and length of hypocotyl and epicotyl was more in transgenic than WT plants after Cd^2+^ stress (Figure 3B–D). Furthermore, Cd^2+^ stress reduced the chlorophyll content in all plants but symptoms of chlorosis were more severe in WT as compared to transgenic plants (Figure 4B–C). Similarly, transgenic lines showed improved roots morphological characteristics and root activity under 7- and 14-days Cd^2+^ stress compared to WT (Figure 5). In plants, cadmium initially accumulates in roots and some Cd^2+^ ions are also transported to shoots. This accumulation of Cd^2+^ causes toxicity that results in growth inhibition and damage to different plant organs [47]. Liu et al. [48] described that high Cd^2+^ accumulation activates MAPK and its transcription factors, resulting in cellular response to overcome Cd^2+^ toxicity. This result supported the positive role of *SlMAPK3* overexpression against Cd^2+^ stress.

Similar to Cd^2+^ stress, drought stress affects many physiological and metabolic processes including photosynthesis, respiration, and overall plant growth. In the current study, *SlMAPK3* overexpression enhanced the tolerance to drought stress in transgenic tomato as reflected by the germination rate and the phenotype of mature plants under drought stress (Figure 7A,B and Figure 8A). Furthermore, transgenic plants showed a reduced leaf water loss rate and improved RWCs as compared to that of WT (Figure 7C,E). Minimization of water loss is essential for the plant to survive under drought conditions. Transpiration, an essential physiological process by which water is transported to leaves and released through stomata, is considered the main pathway of water loss in plants. ABA as a signal molecule observes stresses and activates changes in guard cells to close stomata, thus preventing excessive water loss through transpiration [49]. In our study, ABA up-regulated *SlMAPK3* expression level within 1 h of application (Figure S2A). These results in agreement with previous reports, which showed that *SlMAPK3* and *ZmSIMK1* expressions were up-regulated upon ABA treatment and overexpression of these protein kinases resulted in increased expression of ABA biosynthesis genes [35,50], while silencing of *SlMPK4* and *NtMPK4L* genes resulted in reduced drought tolerance due to enhanced stomatal opening-induced rapid transpiration and subsequent wilting under drought stress [51,52]. These results showed that *SlMAP3* might be involved in ABA-mediated stomatal closure in plants adaptation to drought conditions.

Drought stress causes an imbalance between light capture and utilization and inhibits photosynthetic activity in plants [53]. The inhibition of PSII activity leads to changes in quantum yield due to an imbalance between electron generation and utilization [54]. Furthermore, under drought conditions, chlorophyll content and chlorophyll fluorescence gradually decrease in *A. thaliana* [55]. In line with this, we found that drought stress decreased chlorophyll content and chlorophyll fluorescence in all lines. However, the fluorescence parameters, øPSII, *Fv/Fm, Fv’/Fm’* and qP were more sensitive in WT than in the transgenic lines (Figure 9). These results are in agreement with Ziaf et al. [56] who also observed similar changes in chlorophyll fluorescence in tobacco plants overexpressing a drought responsive gene.

Interestingly, the value of NPQ increased after drought stress and was significantly higher in *SlMAPK3* overexpressed lines (Figure 9E). Plants under water stress efficiently dissipate energy trapped at PSII in the form of heat [57], the dissipation process start after increase in photosystem II S protein (PsbS) level during long-term water stress. PsbS is a small subunit of PSII and plays a key role in energy dissipation [58]. The PsbS protein induces the elevation of NPQ after binding to LHCII trimers with concomitant rearrangements [59,60]. The rise in NPQ in drought stress might also be due to stomata closure that inhibits the availability of carbon dioxide (CO_2_) in the leaf chloroplasts [61], and NPQ has a protective role against photo-inhibition. It can be concluded that *SlMAPK3* overexpression not only increased chlorophyll content but also improved photosynthetic activity compared to WT after drought stress.

Environmental stress causes oxidative damage to different cellular components and ultimately impairs membrane integrity [62,63]. Membrane integrity can be estimated by REL and MDA accumulation [64,65]. In the current study, the stress-induced EL percentage and MDA content were significantly lower in overexpressed plants compared to WT (Figure 4D, Figure 7D and Figure 8C), indicating that the *SlMAPK3* overexpression attenuated membrane damage in transgenic lines. It was previously shown that wheat MAP kinase phosphatase 1 reduced MDA content in *Arabidopsis* after stress [66]. Similarly, *SlMPK7* overexpression in transgenic tomato alleviates stress-induced relative electrolyte leakage and MDA content through activation of the cellular antioxidant systems and stress-associated genes [41].

ROS such as H_2_O_2_ and O_2_^−^, accumulated in plant tissue either cause oxidative damage at high concentration, or function as secondary messengers at low concentrations to mediate essential plant growth and developmental processes. Therefore, plants try to maintain a steady-state of ROS through collaboration between ROS production and elimination [67]. H_2_O_2_ is considered as one of the most stable ROS species and the H_2_O_2_ production is triggered by Cd^2+^ and drought stresses in plants [68,69]. An increase in H_2_O_2_ level was observed in leaves of both WT and transgenic lines under Cd^2+^ and dehydration stresses. However, *SlMAPK3* overexpressing lines accumulated relatively lower levels of H_2_O_2_ than WT (Figure 4E and Figure 8B), which is consistent with previous reports on protein kinase regulated scavenging of ROS during drought stress [70]. Cd^2+^-activated *AtMAPK3* are effectively inhibited by pre-treatment with glutathione (ROS scavenger), further supporting the possible interaction of Cd^2+^ singling, ROS accumulation, and MAPK3 activation [48].

To alleviate ROS levels, plants enhanced the activity of various antioxidant enzymes that contribute to ROS scavenging and detoxification [71,72]. The current study showed that *SlMAPK3* overexpression induced antioxidant enzymes activities in transgenic lines compared to that of WT after Cd^2+^ and drought stresses (Figure 6 and Figure 10). Here, prolonged Cd^2+^ stress inhibited the POD activity in all lines and this decrease might be due to that the of severe Cd^2+^ stress on plants defense mechanisms leading to reduced protein synthesis [73]. Soudek et al. [74] also described the reduction of POD activity under high Cd^2+^ stress in *Sorghum species*. Similarly, *SlMAPK3* overexpression induced up-regulation of the transcript level of *SOD, POD, CAT* and *APX* under stress conditions in transgenic lines, which showed a post-transcriptional effect on the induction and activity of ROS scavenging enzyme. Previous reports have shown that overexpression of different MAPKs cascade genes improves ROS homeostasis through activation of antioxidant systems or modifying transcriptional levels of various stress-associated genes [75,76]. Proline and sugar are important bio-macromolecules that play an essential role in plant development, stress tolerance, improve plant signaling, balancing cell redox status, cellular and osmotic adjustment and scavenging of ROS [77,78,79]. Changes in proline and soluble sugars have often been reported in plants exposed to harsh conditions, however, the current investigation showed that *SlMAPK3* overexpression have role in additive up-regulation of the accumulation of proline and soluble sugar in overexpressed plants, suggesting that MAPK3 also improved the compatible solutes mechanism involved in ROS scavenging. The results are in line with Pan et al. [76], who reported the involvement of MAPKs in the accumulation of proline and soluble sugar during stress conditions. Taken together, these findings suggested that *SlMAPK3* improves stress tolerance by regulating ROS-scavenging mechanism under stress conditions.

In summary, we found that Cd^2+^ and drought affect plant growth and development by altering various physiological and molecular mechanisms. The Cd^2+^ and drought-induced oxidative stress was attributed to an increased accumulation of ROS in tomato plants. *SlMAPK3* overexpression alleviated the toxic effects of Cd^2+^ by improving root morphology and antioxidant activity, whereas elevation of photosynthetic activity, antioxidant system osmoprotectants conferred drought tolerance in transgenic plants. These findings also suggest that there might be an interaction of *SlMAPK3* activation with H_2_O_2_ and ABA in Cd^2+^ and drought stress, respectively. Thus, we conclude that MAPKs-cascade has an important role in enhancing plant tolerance to different abiotic stressors, however, further studies on upstream to downstream kinases are required to better understand the complete pathways for Cd^2+^ and drought tolerance in plants.

## 4. Materials and Methods

### 4.1. Plant Materials, Growth Conditions, and Treatments

In the present study, *Solanum lycopersicum* L. cv. M82, was used to study the expression patterns of *SlMPK3* gene. Tomato seeds were sown in pots containing a mixture of soil, peat, and vermiculite. The seedlings were grown in a growth chamber with a 16/8 h light/dark photoperiod at 25 °C, relative humidity of 70–80% with 200 μmol m^−2^ s^−1^ light intensity.

### 4.2. Tissue Specific Expression Analysis of SlMPK3 Gene

For tissue specific expression analysis, samples of stems, leaves, roots, flowers, fruits, and seeds were harvested from tomato plants. More specifically, root, stem and leaves samples were collected from five-week old plants, whereas fruits at the mature green stage and flowers at anthesis stage were sampled. All the collected samples were initially frozen in liquid nitrogen and stored at −80 °C until isolation of RNA for qRT-PCR analysis.

### 4.3. SlMPK3 Expression Analysis under Abiotic Stress and Hormonal Treatment

Six-week old tomato plants were used to assess the changes in *SlMPK3* expression in response to different abiotic stressors (cadmium, drought, salt and heat,) and hormonal treatments (ABA, SA and MeJA). For the imposition of cadmium stress, seedlings were uprooted, and thoroughly washed with tap water and transferred to full-strength Hoagland’s solution for 48 h and then shifted to a similar solution containing 150 μM Cd^2+^. Meanwhile, a group of seedlings was dehydrated on filter paper to impose drought treatment. For salt stress, seedlings were irrigated with 200 mM NaCl, while heat stress was imposed by keeping the plants at 40 °C in a growth chamber. For hormonal treatment, 100 μM ABA, MeJA or SA solutions were directly sprayed on to the foliar portion, and mock-treated plants were used as the control. Young top leaves of different plants were collected at various time points (0, 1, 3, 6, 12 and 24 h) after the treatments and immediately frozen in nitrogen and stored at −80 °C for RNA isolation.

### 4.4. Abiotic Stress Tolerance Assay in SlMPK3 Overexpressing Lines

T_2_ generation of *SlMPK3* overexpressing (L-4, L-6, and L-7) and WT lines were used for cadmium and drought tolerance assay. For germination assay, seventy five seeds of WT and overexpressing lines per replication were germinated on half-strength MS medium supplemented with or without 100 μM Cd^2+^ and 200 mM mannitol. Germination was counted for seven days and seeds with radical length 2 mm long were considered germinated. Hypocotyl length of the thirty seedlings was measured with ruler from root/shoot junction to the point where cotyledon petioles branched. For cadmium tolerance assay, the uniform size seedlings of wild-type and transgenic lines at the fully expanded three-leaf stage were transferred into containers (60 × 20 × 12 cm) filled with basic Hoagland’s nutrient media for hydroponic cultures. At fully expanded five-leaf stage plants were exposed to cadmium stress, the plants were grown in cadmium free medium for two weeks considered as 0 d stress, while for 7 d stress, plants were grown first for a week without cadmium prior to transfer to stress condition for a week and in 14 d stress condition, plants were immediately transferred to cadmium medium. Cadmium with a concentration of 100 μM was used as CdCl_2_ 2.5 H_2_O from already prepared stock solution in distilled water. The concentration of CdCd^2+^ was used according to the published reports [80,81]. The solution was renewed every week with or without Cd^2+^ for stress and control conditions, respectively. After fourteen days of stress condition, plants were harvested and the experiment was terminated. For molecular and physiological analysis, the harvested leaves were frozen in liquid nitrogen and kept at −80 °C. Roots morphology was studied using “Epson Perfection V700” photo flatbed scanner and root activity, root fresh and dry weight were measured for control and stress-exposed plants.

For drought tolerance evaluation, the uniform-size seedlings of transgenic and WT lines were divided into two groups (control and drought groups). The control group was watered every second day, while drought treatment was imposed by withholding water for 15 days. Fifteen days after stress conditions, the topmost leaves from the tested plant were collected, frozen in liquid nitrogen and stored for further analysis. To determine water loss essay, the top second fully expanded leaves of both transgenic and WT lines were detached and placed on filter papers in the presence of white fluorescent light at room temperature. The harvested leaf samples were weighed after every thirty minutes of interval up to 7 h and measured as a percentage of the control. Chlorophyll fluorescence was examined after 30 min of dark adaptation in the third fully expanded leaves using PAM-2500 Portable Modulated Chlorophyll Fluorometer (Heinz Walz, Effeltrich, Germany). Notably, for this assay, about two months old transgenic and WT plants were subjected to drought conditions for 20 days to assess drought tolerance at the late vegetative stage.

### 4.5. Phylogenetic and Protein Domain Analysis

The amino acid sequences of *SlMAPKs* proteins were gained using NCBI (https://www.ncbi. nlm.nih.gov/) online tool. The phylogenetic tree was generated using MEGA5 software (Pennsylvania State University, State College, USA) by Neighbor-Joining (NJ) method with a Poisson model and replicates test of 1000 bootstrap [82]. The protein domains were identified using the online Single Modular Architecture Tool, SMART (http://smart.embl-heidelberg.de/). All the proteins sequence information used was listed in Table S1.

### 4.6. Vector Construction and Gene Transformation

The full coding sequence of *SlMPK3* was amplified from cDNAs by polymerase chain reaction (PCR) using specific primers (Table S2). The product was cloned into the pMD-18T vector and after sequencing, the correct pMD-18T-*SlMPK3* was double digested using *Bam*HI and *Sac*I restriction sites. The product was ligated into plant expression vector pVBG2307 and the resulting construct was transformed into *Agrobacterium tumefaciens* strain GV3101. Standard leaf protocol was used for transformation of tomato [83]. Sterile cotyledons pieces from 10–12 days seedlings were transferred to *Agrobacterium* suspended MS solution (OD_600_ = 0.5) containing acetosyringone for 15 min and then incubated for 48 h on MS media without antibiotics. The explants were transferred to MS selection media containing 50 mg L^−1^ kanamycin and 200 mg L^−1^ carbenicillin with proper amount of hormones. The shoots developed from the calli were shifted to rooting media containing 30 mg L^−1^ kanamycin and 200 mg L^−1^ carbenicillin. The kanamycin resistance plants were further confirmed by genomic PCR using *nptII* forward and *SlMAPK3* reverse primers. Seeds were harvested from inbred lines and transgenic T_2_ lines were used for further experiments.

### 4.7. RNA Isolation and Real-Time qRT-PCR

Total RNA was isolated using Trizol reagent (Invitrogen, Carlsbad, CA, USA) and cDNA was synthesized using Takara Prime Script RT reagent Kit (Takara, Dalian, China). Thermo cycler iQ5 Real-Time PCR Detection System (BIO-RAD Corp., Hercules, CA, USA) and SYBR Premix Ex TaqTM (Takara, Dalian, China) were used following manufacturer guidelines to run qRT-PCR. The PCR conditions were: 95 °C for 1 min, followed by 40 cycles of 95 °C for 10 s, 55 °C for 10 s and 72 °C for 20 s. Tomato actin gene was used as an internal control. All the primers used in the experiment are listed in Table S2. The 2^−ΔΔCT^ method was applied to analyze the PCR data [84].

### 4.8. Root Activity Assay

Root activity of the stressed and un-stressed plants was measured using triphenyltetrazolium chloride (TTC) method [85]. Roots from stressed and un-stressed plants were collected and washed with distilled water. 0.2 g of roots tips were cut (1 cm) and dried on clean filter papers. The dried tips were placed in tubes and soaked with a mixture of TTC (1%) and phosphate buffer (0.l M-pH 7.0) solution at 37 °C for 2 h in dark. The reaction was stopped by adding of sulfuric acid (5%, H_2_SO_4_) after 2 h incubation. The roots tip were collected and dried again on filter papers. Roots were ground with 5 mL ethyl-acetate and certain amount quartz sand in mortar and pestle to extract the triphenyl formazan (TTF). The final volume of the extract was diluted with ethyl-acetate to 10 mL. Absorption was taken at 485 nm and reduced TTC was calculated from the standard curve. TTC reduction intensity (mg g^−1^ h^−1^) = TTC reduced/FW h, where FW and h is root fresh weight and incubation time respectively.

### 4.9. Measurement of Leaf Water Content and Electrolyte Leakage

RWC was measured as described by Shekhawat et al. [86]. Leaves from WT and transgenic lines were harvested and immediately weighed for fresh weight (FW). Next, the leaves were floated on de-ionized water at 4 °C for overnight to measure the turgid weight (TW). For dry weight (DW) analysis, the leaves were dried in a hot oven for 2 days at 60–62 °C. Water content was calculated as: RWC = (FW − DW)/(TW − DW) × 100. Electrolyte leakage was determined according to the protocol of Compose et al. [87]. The fourth fully expanded leaves of WT and transgenic plants were excised from well-watered and drought-stressed plants. The leaves were first rinsed with distilled water and then fifteen leaf discs (diameter, 8 mm) were punched. The leaf discs were immersed in 25 mL distilled water containing tubes and were shaken at 300× *g* at 25 °C for 4 h in dark. After 4 h of incubation, the electrolyte leakage (EC1) of the solutions was recorded. The leaf discs solutions were then boiled for 20 min, cooled down at room temperature and then electrolyte leakage (EC2) of the solutions was recorded again. Relative electrolyte leakage (%) was measured using a conductivity detector and results obtained as: (EC1/EC2) × 100.

### 4.10. Determination of Chlorophyll, Soluble Sugar and Proline Content

Chlorophyll content was determined in leaves using 80% acetone. The fresh leaves from stressed and control plants were incubated overnight in 80% acetone and the leaf pigments were retained. The absorbance was taken at 663 and 646 nm and chlorophyll content were measured in milligrams per gram FW [88]. Soluble sugar content was analyzed with anthrone colorimetry [89]. About 0.2 g leaf samples (small pieces) were boiled in 10 mL distilled water for 30 min. The liquid extract was separated in 25 mL tube and repeated again. The extract was diluted to 25 mL final volume. Sample extract (0.5 mL) was mixed with distilled water (1.5 mL), 2% (*w/v*) anthrone ethyl acetate reagent (0.5 mL) and 100% H_2_SO_4_ (5 mL). The mixture was boiled again for 10 min and after cooling, absorbance was recorded at 630 nm. The amount of soluble sugar was calculated from a standard curve of known amounts of glucose. Proline was measured as the protocol described by Zhang et al. [90]. Leaf sample (0.3 g) was boiled with 3% sulphosalicylic acid (5 mL) for 10 min at 100 °C. The mixture was filtered and filtrate (2 mL) was mixed with acid ninhydrin reagent (2 mL) and glacial acetic acid (2 mL). The combined mixture was heated again for 40 min at 100 °C and reaction was terminated in an ice bath. Finally, cooled mixture was treated with toluene (5 mL) and the absorbance of upper organic phase was determined at 520 nm. Proline content was measured from the standard curve of known amounts of proline.

### 4.11. Determination of H_2_O_2_ Content and Histochemical Detection of H_2_O_2_

The H_2_O_2_ content was measured according to Sergiev et al. [91]. Leaf samples (0.5 g) were homogenized in ice bath with trichloroacetic acid (TCA) 0.1% (*w/v*) solution. The homogenate was centrifuged for 15 min at 12,000× *g* and the supernatant was added to 1 M potassium iodide and 10 mM potassium phosphate buffer pH 7.0. The solution was briefly mixed and absorbance was recorded at 390 nm. The H_2_O_2_ content was measured from the standard curve. Standard solutions for H_2_O_2_ curve were prepared by dilution of 100 µM H_2_O_2_ and 0.1% TCA in ration of 0:1, 0.1:0.9, 0.2:0.8, 0.4:0.6, 0.6:0.4, 0.8:0.2, and 1:0 mL in 3 mL of potassium phosphate buffer and potassium iodide solution. For in situ visualizations of H_2_O_2_ accumulation, leaflets of the second fully expanded leaves from stressed and control plants of WT and transgenic lines were used. The leaves were dipped in 1 mg mL^−1^ DAB, pH 3.8 and incubated for 8 h in dark condition. For removal of chlorophyll, the DAB stained leaves were shifted to absolute ethanol and heated in a boiling water bath for 10 min [92].

### 4.12. Assay of Lipid Peroxidation and Antioxidant Enzymes Activity

Lipid peroxidation was estimated by analyzing MDA content according to Campos et al. [87]. About 0.5 g of leaf sample was ground in sodium phosphate buffer (pH 7.8) having 1% polyvinyl pyrrolidone (PVP) on an ice bath. The extract was centrifuged at 12,000× *g*, for 20 min at 4 °C and the supernatant was mixed with 5% TCA having 0.5% 2-thiobarbituric acid (TBA) and boiled for 20 min at 95 °C water bath. The solution was cooled, centrifuged at 5000 g for 10 min and then the absorbance was measured at 600, 532 and 450 nm. Antioxidant enzymes activities were analyzed according to the protocol described by Guo et al. [93]. For SOD activity assay, the crude enzyme was further diluted with water containing 130 mM methionine, 0.1 mM EDTA-Na_2_, 0.75 mM nitrogen blue tetrazolium (NBT), 0.02 mM riboflavin, and 0.05 mM phosphate buffer. Reaction mixture without enzymes was used as a control. All the reaction tubes and one control tube was placed in a light incubator at 4000 lux illumination for 20 min and covered with black fabric immediately, while another control tube was kept in dark. The activity was measured at 560 nm. For POD activity, reaction mixture contained enzyme extract, 0.2% guaiacol and 0.3% H_2_O_2_ diluted with 0.2 mM phosphate buffer (pH 7.0). The kinetic was recorded at 470 nm wavelength for 3 min reaction time. CAT activity was examined by observing the decrease in the activity of the enzyme. The extraction enzyme was mixed with 0.3% H_2_O_2_ diluted with 0.2 mM phosphate buffer (pH 7.0) and activity was measured at 240 nm for 3 min every 30 sec. APX activity was analyzed according to the protocol of Jiang and Zhang [94]. The reaction mixture contained 0.1 mM H_2_O_2,_ 50 mM potassium phosphate buffer (pH 7.0), 0.5 mM ascorbate (ASC) and enzyme extract of 100 µL. The reaction was started by adding enzyme solution and activity was recorded at 290 nm. All of the spectrophotometric measurements were performed on a UNICO WFZ 3802H UV/VIS spectrophotometer (UNICO, Shanghai, China).

### 4.13. Statistical Analysis

SPSS 20.0 software (IBM, Armonk, New York, USA) was used for statistical analysis. A significant difference between WT and transgenic lines were determined using least significance difference (LSD) test. Means ± SE were calculated from the average of three biological replicates. Asterisks * at *p* < 0.05 and ** at *p* < 0.01 show different significance levels.

## Supplementary Materials

The following are available online. Figure S1, Phylogenetic and structural analysis based on the amino acid sequences of *Solanum lycopersicum SlMAPKs*; Figure S2, Expression patterns of *SlMAPK3* after different hormonal treatments; Figure S3, Confirmation of transgenic plants. Table S1, The protein sequence information used for phylogenetic tree construction and protein domain; Table S2, Primers used in the study.
