# Supplementary material for: Overexpression of a Mitogen-Activated Protein Kinase SlMAPK3 Positively Regulates Tomato Tolerance to Cadmium and Drought Stress
Source: Molecules. 2019 Feb 3;24(3):556. doi: 10.3390/molecules24030556 (PMC6385007; doi:10.3390/molecules24030556)
Supplement: Supplementary file 1 [file molecules-24-00556-s001.pdf]

Table S1. The protein sequence information used for phylogenetic tree construction and proteins domain

Gene Name	Solgenomics accession ID	Gene accession No.	Protein ID
SlMPK1	Solyc12g019460.2	NM_001247082.2	NP_001234011.2
SlMPK2	Solyc08g014420.3	NM_001247426.1	NP_001234355.1
SlMPK3	Solyc06g005170.3	NM_001247431.2	NP_001234360.1
SlMAPK4	Solyc11g072630.2	HM367593.1	ADK38705.1
SlMAPK5	Solyc01g094960.3	HM036493.2	ADH43227.2
SlMAPK6	Solyc05g049970.3	JF791807.1	AEH42637.1
SlMAPK7	Solyc08g081490.2	JF791808.1	AEH42638.1
SlMAPK8	Solyc02g084870.3	HM189670.1	ADJ95343.1
SlMAPK9	Solyc04g080730.3	HM245320.1	ADK13095.1
SlMAPK10	Solyc10g007500.3	JF795445.1	AEH42667.1
SlMAPK11,	Solyc07g062080.3	JF795446.1	AEH42668.1
SlMAPK12	Solyc07g056350.3	JF795447.1	AEH42669.1
SlMAPK13	Solyc01g080240.3	JF795448.1	AEH42670.1
SlMAPK14	Solyc04g007710.3	JF795449.1	AEH42671.1
SlMAPK15	Solyc05g008020.3	JF795451.1	AEH42673.1
SlMAPK16	Solyc12g040680.2	JF795450.1	AEH42672.1

Table S2. Primers used in the study

Genes	Primer sequences (5'-3')	
	Forward	Reverse
SlMPK3	CGGGATCCATGGTTGATGCTAATATGGGTG	GGCGCGAGCTCTTAAGCATATTCAGGATTCAACG
NPTII	TCTCATGCTGGAGTTCTTCGC	GTCACCGACTTGAGCCATTTG
qSlMPK3	CTCATCCCTACCTCGCAAAG	TCAGGATTCAACGCCAAAGC
qSlSOD	ACTTGCTCCTGGACTTCACG	TCGGCATTGGCCACTATGTT
qSlPOD	GGAATGTGCCAACTGGTAGA	TGATGAGCAACGAGACACTC
qSlCAT	TGGAAGCCAACTTGTGGTGT	ACTGGGATCAACGGCAAGAG
qSlAPX	TGTGAGCGAGGAGTACCTCA	TGTGCTAGCTCCGCTTTGAA
qSlActin	TGGTCGGAATGGGACAGAAG	CTCAGTCAGGAGAACAGGGT

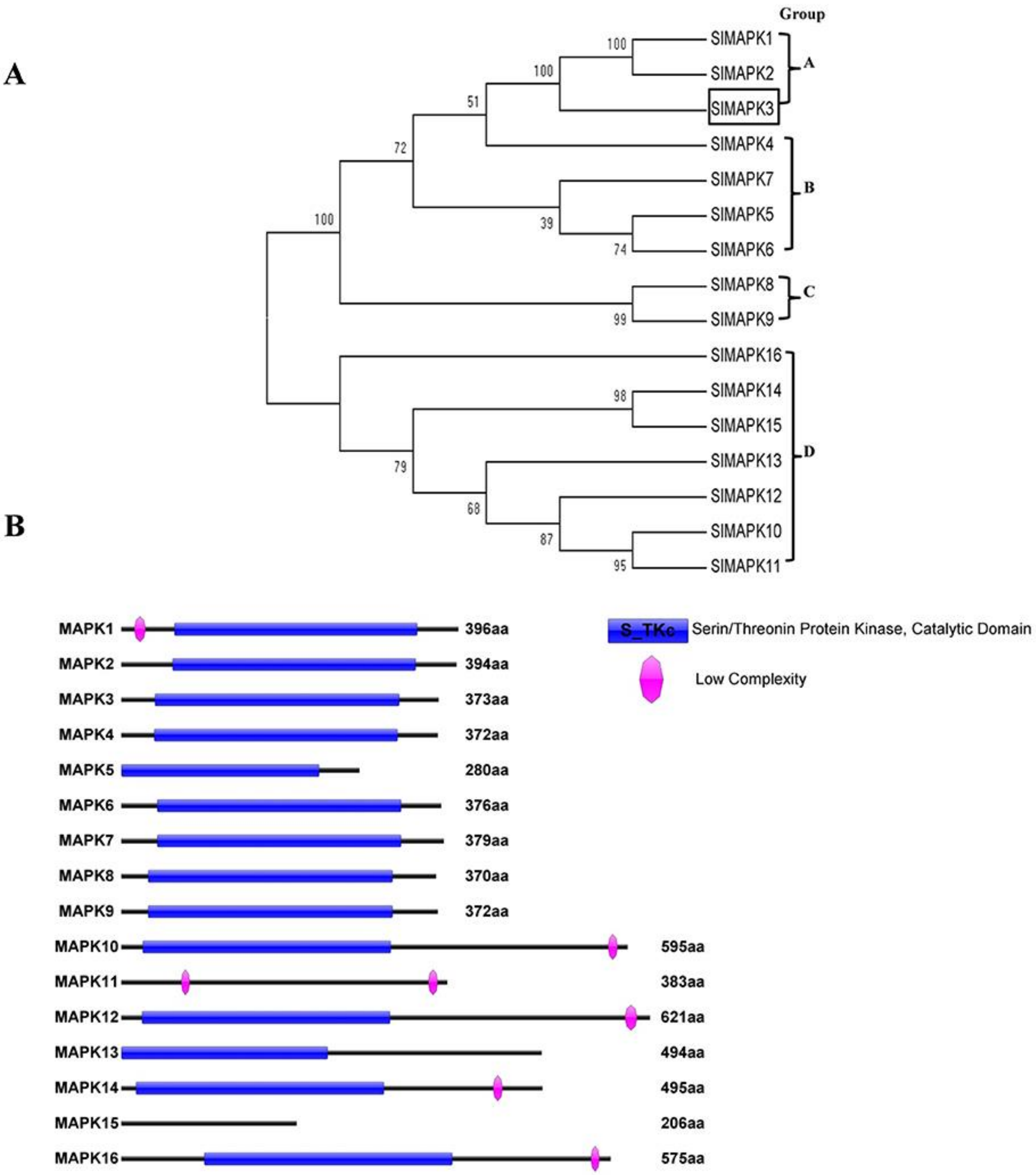

Figure S1. Phylogenetic and structural analysis based on the amino acid sequences of *Solanum lycopersicum SlMAPKs.* (A) Phylogenetic tree, the tree was generated using MEGA5 program by the neighbor-joining method. Bootstrap supports from 1000 replicates are indicated at each branch and (B) Domain position of *SlMAPKs* in tomato. Domains are indicated as boxes in different colors.

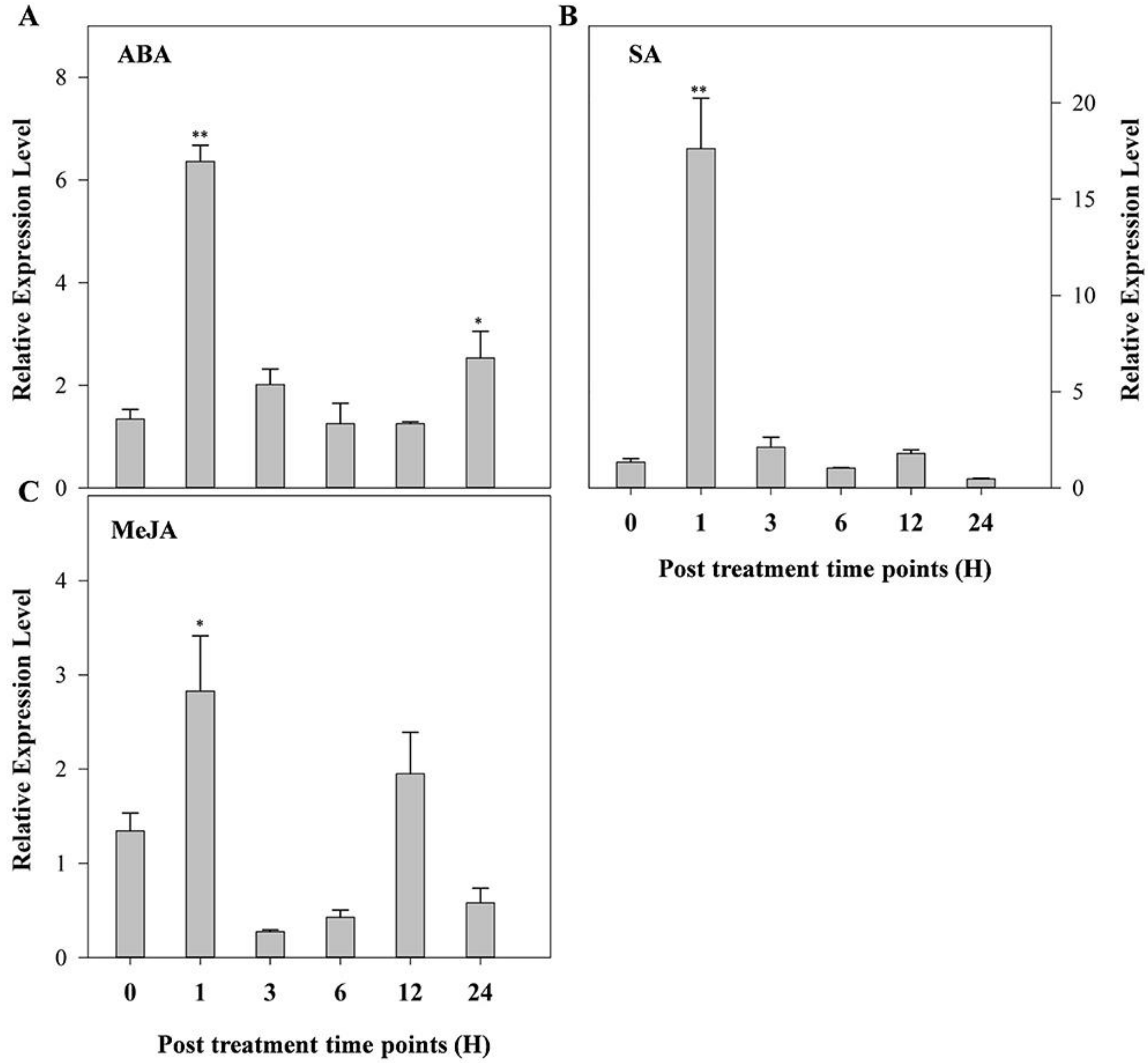

Figure S2. Expression patterns of *SlMAPK3* after different hormonal treatments. Expression patterns of *SlMAPK3*gene under different exogenous hormones. (A) ABA -100 μM, (B) SA -100 μM and (C) MeJA -100 μM for 0, 1, 3, 6, 12, and 24h. Zero (0) represents seedlings without any treatment. The tomato actin gene was used as the internal control for normalization. Data represent mean ±SE of three biological replicates.

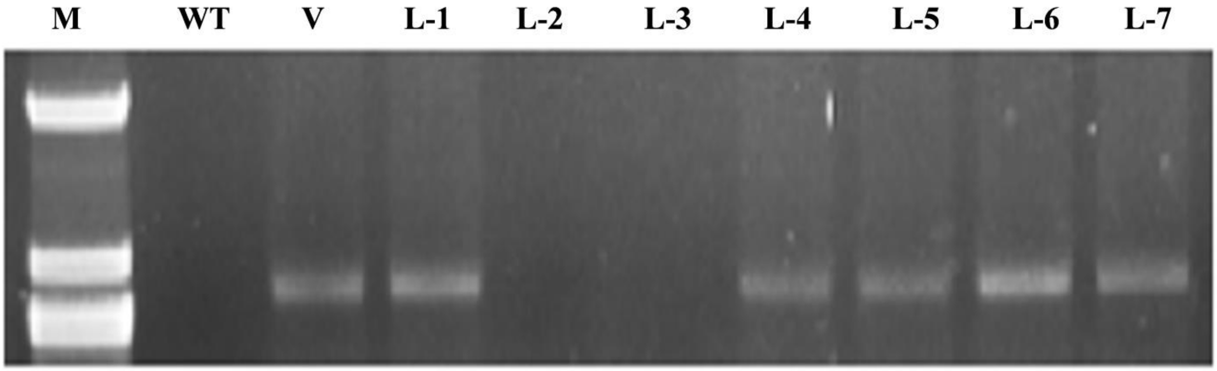

Figure S3. Confirmation of transgenic plants. PCR detection of NPTII fragments in T_0 transgenic plants. M: DNA marker, WT: Wild-type plants, V：pVBG2307-MPK3 vector and L1~7: 7 lines of T_0 transgenic plants.
